# The Principles of Botany

**Published:** 1840-07

**Authors:** 


Art. VIII.—*The Principles of Botany; Structural, Functional, and Systematic. Condensed and immediately adapted to the Use of Students in Medicine.* By W. Hughes Willshire, m.d., Lecturer on Botany at Charing Cross Hospital.—*London*, 1840. 12mo, pp. 332.

"It was not," says the preface, "on account of any deficiency of works introductory to the study of botany that the author was induced to make public this little book, but because he thought he could present to the student of medicine *a more condensed view of the first principles of the science, combined with circumstances, and illustrated in a manner which would more immediately interest him*, than was done by any mere single text-book with which the author was acquainted." In this opinion we are, on the whole, inclined to agree. Without anything very original, either in facts or in the interpretation of them, and in some instances with what appears to us rather too free a use of works which have a similar object with his own, Dr. Willshire has produced a little treatise which will, we doubt not, from its clearness and simplicity, be acceptable as well as useful to those for whom it is designed. In the anatomical and physiological portion he has availed himself of the most recent information at his command, and interwoven it well with that of older date. We regret, however, that he should have so completely neglected the cryptogamia in this portion of the work; and that he should have adopted in his systematology the primary divisions of Decandolle, since these have been proved to be founded on erroneous principles.

